# Factors influencing the mental health of an ethnically diverse healthcare workforce during COVID-19: a qualitative study in the United Kingdom

**DOI:** 10.1080/20008066.2022.2105577

**Published:** 2022-08-09

**Authors:** Irtiza Qureshi, Mayuri Gogoi, Amani Al-Oraibi, Fatimah Wobi, Jonathan Chaloner, Laura Gray, Anna L Guyatt, Osama Hassan, Laura B. Nellums, Manish Pareek, On behalf of the UK-REACH Collaborative Group

**Affiliations:** a Lifespan and Population Sciences, School of Medicine, University of Nottingham, Nottingham, UK; b Department of Respiratory Sciences, University of Leicester, Leicester, UK; c Department of Health Sciences, University of Leicester, Leicester, UK; d Department of Infection and HIV Medicine, University Hospitals of Leicester NHS Trust, Leicester, UK

**Keywords:** Ethnic minority, healthcare, workforce, mental health, COVID-19, anxiety, trauma, stress, Minoría étnica, cuidadores de salud, fuerza laboral, salud mental, COVID-19, ansiedad, trauma, estrés, 少数族裔, 医护, 劳动力, 心理健康, COVID-19, 焦虑, 创伤, 压力

## Abstract

**Background:** Healthcare workers (HCWs) have been reported to be experiencing a deterioration in their mental health due to COVID-19. In addition, ethnic minority populations in the United Kingdom are disproportionately affected by COVID-19. It is imperative that HCWs are appropriately supported and protected from mental harm during the pandemic. Our research aims to add to the evidence base by providing greater insight into the lived experience of HCWs from diverse ethnic backgrounds during the pandemic that had an impact on their mental health.

**Methods:** We undertook a qualitative work package as part of the United Kingdom Research study into Ethnicity And COVID-19 outcomes among Healthcare workers (UK-REACH). As part of the qualitative research, we carried out 16 focus groups with a total of 61 HCWs between December 2020 and July 2021. The aim of the study was to explore topics such as their experiences, fears and concerns, while working during the pandemic. The purposive sample included ancillary healthcare workers, doctors, nurses, midwives and allied health professionals from diverse ethnic backgrounds to ensure inclusion of underrepresented and disproportionately impacted individuals. We conducted discussions using Microsoft Teams. Recordings were transcribed and thematically analysed.

**Results:** Several factors were identified which impacted on the mental health of HCWs during this period including anxiety (due to inconsistent protocols and policy); fear (of infection); trauma (due to increased exposure to severe illness and death); guilt (of potentially infecting loved ones); and stress (due to longer working hours and increased workload).

**Conclusion:** COVID-19 has affected the mental health of HCWs. We identified a number of factors which may be contributing to a deterioration in mental health for participants from diverse ethnic backgrounds. Healthcare organisations should consider developing strategies to counter the negative impact of these factors, including recommendations made by HCWs themselves.

## HIGHLIGHTS

- HCWs and ethnic minorities are experiencing deterioration in their mental health due to COVID-19.
- Little is known about the lived experience of HCWs from diverse ethnic backgrounds and their mental health during the pandemic.
- This research highlights relevant factors such as anxiety (due to inconsistent protocols and policy), fear (of infection), trauma (due to increased exposure to severe illness and death), guilt (of potentially infecting loved ones) and stress (due to longer working hours and increased workload).

## Abstract (es)

**Antecedentes:** Se ha informado que los trabajadores de la salud (HCW, por sus siglas en inglés) están experimentando un deterioro en su salud mental debido al COVID-19. Además, las poblaciones de minorías étnicas en el Reino Unido se ven afectadas de manera desproporcionada por el COVID-19. Es imperativo que los trabajadores de la salud reciban el apoyo adecuado y estén protegidos de afecciones mentales durante la pandemia. Nuestra investigación tiene como objetivo aumentar la base de evidencia al proporcionar una mayor comprensión de la experiencia vivida por los trabajadores de la salud de diversos orígenes étnicos durante la pandemia que tuvieron un impacto en su salud mental.

**Metodología:** Llevamos a cabo un paquete de trabajo cualitativo como parte del estudio de investigación del Reino Unido sobre los resultados de la etnicidad y el COVID-19 entre los trabajadores de la salud (UK-REACH). Como parte de la investigación cualitativa, llevamos a cabo 16 grupos focales con un total de 61 Trabajadores de la Salud entre diciembre de 2020 y julio de 2021. El objetivo del estudio fue explorar temas como sus experiencias, miedos y preocupaciones, mientras trabajaban durante la pandemia. La muestra intencional incluyó trabajadores auxiliares de la salud, médicos, enfermeras, matronas y profesionales de la salud asociados de diversos orígenes étnicos para garantizar la inclusión de personas subrepresentadas y desproporcionadamente afectadas. Llevamos a cabo debates utilizando Microsoft Teams. Las grabaciones fueron transcritas y analizadas temáticamente.

**Resultados:** Se identificaron varios factores que afectaron la salud mental de los trabajadores de la salud durante este período, incluida la ansiedad (debido a protocolos y políticas inconsistentes); miedo (de infección); trauma (debido a una mayor exposición a enfermedades graves y muerte); culpa (de infectar potencialmente a los seres queridos); y estrés (debido a jornadas laborales más largas y mayor carga de trabajo).

**Conclusión:** COVID-19 ha afectado la salud mental de los trabajadores de la salud. Identificamos una serie de factores que pueden estar contribuyendo al deterioro de la salud mental de los participantes de diversos orígenes étnicos. Las organizaciones de atención médica deben considerar el desarrollo de estrategias para contrarrestar el impacto negativo de estos factores, incluidas las recomendaciones hechas por los propios trabajadores de la salud.

## Abstract (zh)

**背景：**据报道，医护人员 (HCW) 的心理健康状况由于 COVID-19正在恶化。此外，英国的少数族裔人口受到 COVID-19 的影响尤为严重。HCW 在疫情期间得到适当的支持和保护并且免受精神伤害是很有必要的。我们的研究旨在通过更深入地了解来自不同种族背景的HCW在疫情期间对其心理健康产生影响的生活经历，增加循证基础。

**方法：**作为英国医护工作者种族和 COVID-19 结果研究 (UK-REACH) 的一部分，我们进行了定性工作包。作为定性研究的一部分，我们在 2020 年 12 月至 2021 年 7 月期间开展了 16 个包含 61 名医护人员的焦点小组。研究目的是探讨其在疫情期间工作时的经历、恐惧和担忧等主题。目标样本包括来自不同种族背景的辅助医护工作者、医生、护士、助产士和联盟医护专业人员，以确保纳入代表性不足和受影响不成比例的个体。我们使用微软团队进行了讨论。对录音进行了转录和主题分析。

**结果：**确定了在此期间影响医护人员心理健康的几个因素，包括焦虑（由于不一致的协议和政策）；害怕（感染）；创伤（由于更多地暴露于重大疾病和死亡中）；内疚（可能感染亲人）；和压力（由于更长的工作时间和增加的工作量）。

**结论：**COVID-19 影响了医护人员的心理健康。我们确定了许多可能导致来自不同种族背景参与者心理健康恶化的因素。医护组织应考虑制定策略来应对这些因素的负面影响，包括医护人员自己提出的建议。

## 1. Introduction

Recent evidence suggests that the Coronavirus disease 2019 (COVID-19) pandemic is contributing to increased levels of stress, anxiety and trauma for various populations in the United Kingdom (UK) (Holmes et al., 2020). One of these affected populations is healthcare workers (HCWs) (Gogoi et al., 2021), with evidence that previous pandemics have had a significant negative impact on the mental health of this population (Salazar, Pablo, Vaquerizo-serrano, Catalan, & Arango, 2020). Some international studies have gone further to seek out any linkages between the psychological impact of previous pandemics such as Severe acute respiratory syndrome (SARS) or middle east respiratory syndrome (MERS) on HCWs, in relation to the COVID-19 pandemic (Huang et al., 2021; Chua et al., 2004).

Although previous research has shown that occupational surveys can report higher levels of mental health distress than general population surveys (Goodwin et al., 2013) there is an increasing body of quantitative evidence to suggest that HCWs are also experiencing poor mental health outcomes as a result of the COVID-19 global pandemic (Vizheh et al., 2020). One research study in Italy with over 2000 HCWs revealed that the majority of participants reported COVID-related traumatic experiences at work and 53.8% (95% CI 51.0%–56.6%) displayed symptoms of post-traumatic distress; moreover, 50.1% (95% CI 47.9%–52.3%) showed symptoms of clinically relevant anxiety and 26.6% (95% CI 24.7%–28.5%) symptoms of at least moderate depression (Lasalvia et al., 2021). International studies have found increased psychological burden on HCWs during this pandemic, identifying specific factors that may be contributing to their poorer mental health (Khajuria et al., 2021; Koontalay, Suksatan, Prabsangob, & Sadang 2021).

In the UK, there is an emerging evidence base that demonstrates similar experiences and outcomes within the general population (Pierce et al., 2020) and in particular, HCWs. This emerging research suggests there is a ‘mental health crisis’ for HCWs in the UK (Melbourne et al., 2022). Governmental reports have also acknowledged the additional psychological burden on UK HCWs during the pandemic (PHE, 2020a; House of Commons, 2021). Additionally, an online survey of over 1000 UK HCWs during the first wave of the pandemic found nearly 58% of respondents met the threshold for a clinically significant mental disorder (PTSD = 22%; anxiety = 47%; depression = 47%) (Greene et al., 2021). Furthermore, drivers behind some of these results seemed to revolve around respondents’ fear of infecting others, access to PPE, and redeployment to areas with high exposure to COVID-19.

Alongside the consideration of HCWs, recent research has highlighted that certain groups in society such as those belonging to ethnic minority backgrounds have been disproportionately affected by the pandemic. For example, figures from the Office for National Statistics (ONS) highlighted that ethnic minority populations in the UK are experiencing higher death rates and poorer health outcomes than white ethnic groups (ICNARC, 2020). While ethnic minority groups make up 14% of the UK population (Office for National Statistics. Ethnicity and National Identity in England and Wales, 2012), they disproportionately represent 34% of COVID-19 cases admitted to critical care (ICNARC, 2020). We also know that this overrepresentation applies to both the general population as well as the healthcare workforce (Khunti, Singh, Pareek, & Hanif, 2020). Individuals from ethnic minority backgrounds compose 19.1% of the National Health Service (NHS) workforce in England (WRES, 2019), yet they made up 63% of COVID-19 related healthcare staff deaths in the initial stages of the pandemic (Razaq, Harrison, Karunanithi, Barr, & Asaria, 2020; Cook, Kursumovic, & Lennane, 2020).

In addition, there is evidence that people belonging to ethnic minority backgrounds have experienced poorer mental health outcomes compared to the general population, due to longstanding inequities in accessing appropriate services (Bignall, Jeraj, Helsby, & Butt, 2019; Bhui et al., 2003; Bhui, Warfa, Edonya, McKenzie, & Bhugra, 2007). Unfortunately, recent research suggests that this inequity and resulting disproportionate impact on mental health may be persisting during COVID-19 (Chui et al., 2021; Kapilashrami & Bhui, 2020; Jaspal & Lopes, 2021).

It is imperative that HCWs (including ethnic minority staff) are appropriately supported and protected from mental harm during the pandemic, as the World Health Organization (WHO) have rightly identified them as ‘our most valuable resource for health’ (Joseph & Joseph, 2016). It will take some time to determine the overall impact of the pandemic, however we need to know more about what factors are contributing to poor mental health and driving disparities within the diverse healthcare workforce, which has been under-examined in the predominantly quantitative research to date. It is also important that those employing ethnic minority HCWs consider their particular context in order to better protect their mental health, retain them and reduce attrition, and professionally support them during and after the COVID-19 pandemic. This is particularly relevant in the context of the extensive research reporting the discrimination and disadvantage that some minority ethnic staff have faced leading up to the pandemic (Allan, Larsen, Bryan, & Smith, 2004; WRES, 2014; Kline, Naqvi, Razaq, & Wilhelm, 2017; Brathwaite, 2018).

While observational studies assessing mental health outcomes among HCWs have been undertaken, qualitative, in-depth studies exploring the factors influencing mental health of HCWs – particularly those from diverse ethnic backgrounds - are scarce (Koontalay et al., 2021; Liberati et al., 2021). This research is part of the largest investigation into COVID-19 and its impact on ethnically diverse HCWs in the UK. Our paper aims to add to the qualitative evidence base on this subject, not to provide diagnostic assessments, but rather to fill this gap by providing greater insight into the lived experience of HCWs from diverse ethnic backgrounds during the pandemic that had an impact on their mental health.

## 2. Methods

### 2.1. Study design and setting

We conducted a qualitative sub-study as part of the wider United Kingdom Research study into Ethnicity and COVID-19 outcomes among HCWs (UK-REACH). This sub-study was specifically designed to explore in-depth HCWs’ experiences, and their perceptions of risks, fears and concerns. The methods utilised for this study, including design, data collection and analysis are further detailed in the relevant protocol (Gogoi et al., 2021).

### 2.2. Study population

We included adult (≥16 years of age) HCWs with experience of working in healthcare settings during COVID-19, including both clinical and ancillary staff, in England, Wales, Scotland and Northern Ireland. HCWs were invited to take part and purposively sampled to include workers from various staff grades, job roles, age, sex, ethnicity, migration status and UK nations, to reflect demographics of the UK workforce and include underrepresented groups. Recruitment was through invitation emails sent out via NHS Trusts, private health contractors, professional bodies, partner organisations, Twitter advertisement and through the Professional Expert Panel (PEP) and UK-REACH stakeholder group (STAG). Participant information sheets containing study details were shared with prospective consenting participants before the start of study procedures. Focus group discussions were conducted between November 2020 and July 2021, but these were not diagnostic in nature and rather aimed to elicit rich descriptions of HCWs’ experiences of working during the pandemic which had an impact on their mental health. As part of a rapid response to disseminate urgently needed evidence, this paper reports data exclusively from the focus groups conducted as part of the overall study.

### 2.3. Data collection

Due to existing restrictions on travel and social distancing, the study was conducted remotely, and all processes including, recruitment, consent, and data collection were completed online. Focus groups were conducted through Microsoft Teams by the research team (IQ, MG, FW, AAO, LB). Key topics covered are included in the topic guide areas included as supplementary material (Appendix). Focus groups were utilised to explore HCWs experiences and opinions in a shared environment which would bring out the similarities and differences in experiences and provide greater breadth to the data. A topic guide was developed in consultation with the UK-REACH PEP and STAG, the public engagement and stakeholder engagement groups respectively.

The final topic guide areas are attached as supplementary material. Discussions took approximately 1.5 hours, with group sizes varying from 2 to 7 members. Following their participation, a £20 gift voucher was given to the participants in recognition of their contribution to the research. Discussions were conducted by the research team who represent a range of ethnicities. The team made up of both male and female members are all trained qualitative researchers and culturally competent in working with diverse ethnic groups. Interviews were recorded with permission, transcribed, anonymised prior to analysis, and checked for accuracy by the research team.

### 2.4. Analysis

Data were analysed using thematic analysis (Braun & Clarke, 2006), following the steps outlined by Braun and Clarke. Coding was carried out inductively to generate codes describing HCWs’ experiences and the related impact on their mental health. Initial coding was carried out independently by four researchers. A coding framework was then developed iteratively through discussion with all authors to generate themes. Thus, generation of these involved a collaborative, organic, and iterative process of developing codes and themes (Braun & Clarke, 2021). The final themes were decided upon following this deep engagement with the data, and a process of reflexive interpretation and interrogation of latent meaning. This point was reached after the research team felt no new themes were identified from the iterations of this process, and all the team members were in agreement with the generated themes. This was an interpretative judgment based on the researchers immersion in the data, independent processes of reflexivity, and the wider purpose of the analysis. Constant interaction with and immersion in the data, independent coding, consultation with the literature (Maher, Hadfield, Hutchings, & de Eyto, 2018), triangulation (e.g. with researcher notes), and active reflexivity were used to strengthen the rigour and trustworthiness of the data (Johnson, Adkins, & Chauvin, 2020).

The process of active reflexivity involved individually and as a group reflecting how our backgrounds and lived experiences might influence our generation and interpretation of data and themes. The researchers come from backgrounds including as ethnic minorities (Black, South Asian, Middle Eastern, and white), migrants, and healthcare workers. The team is made up of both male and female trained qualitative researchers. Their academic and professional backgrounds include public health, health policy, anthropology, social work, medicine, and pharmacy. Because of these backgrounds, the researchers often felt they had shared lived experience with the individuals they were interviewing. This was important to reflect on to interrogate any assumptions made based on their own shared experiences and to proactively seek to identify not only similarities, but also divergences. In addition, the diverse backgrounds of the research team enabled a range of lived experience to be brought to the interpretative analysis, enabling similar diversity, nuance, and richness in the generation of themes.

### 2.5. Ethics

Ethical approval has been received from the London-Brighton & Sussex Research Ethics Committee of the Health Research Authority (Ref No 20/HRA/4718). All participants gave written informed consent.

## 3. Findings

### 3.1. Demographic data

Sixteen focus groups were carried out with a total of 61 participants. Discussions had a duration of about 1.5 hours, with group sizes varying from 2 to 7 members. The details of participants are provided in Table 1. The first column in the table details the variables for participants, the second column shows the sample number, and the final column provides the sample percentage.

**Table 1.** Demographic characteristics of participants.

Characteristics	Sample (*n* = 61)	% of Sample
Male	26	43
Female	35	57
Other	0	0
Age, median (IQR)	46 (22–69)	
Asian	22	36
Black	15	25
Mixed	6	10
White	17	28
Other	1	1
Doctors	13	21
Nurses & Midwives	12	20
Allied Health Professionals*	15	25
Ancillary Health Workers**	21	34
England	52	85
Scotland	1	1
Wales	0	0
Northern Ireland	5	8
Unknown	3	6

*Also Includes dentists, pharmacists, healthcare scientists, ambulance workers and those in optical roles.

** Includes those in administrative, or other non-clinical roles (e.g housekeeping/security/maintenance etc).

### 3.2. Factors impacting on the mental health of HCWs

**Figure 1.**
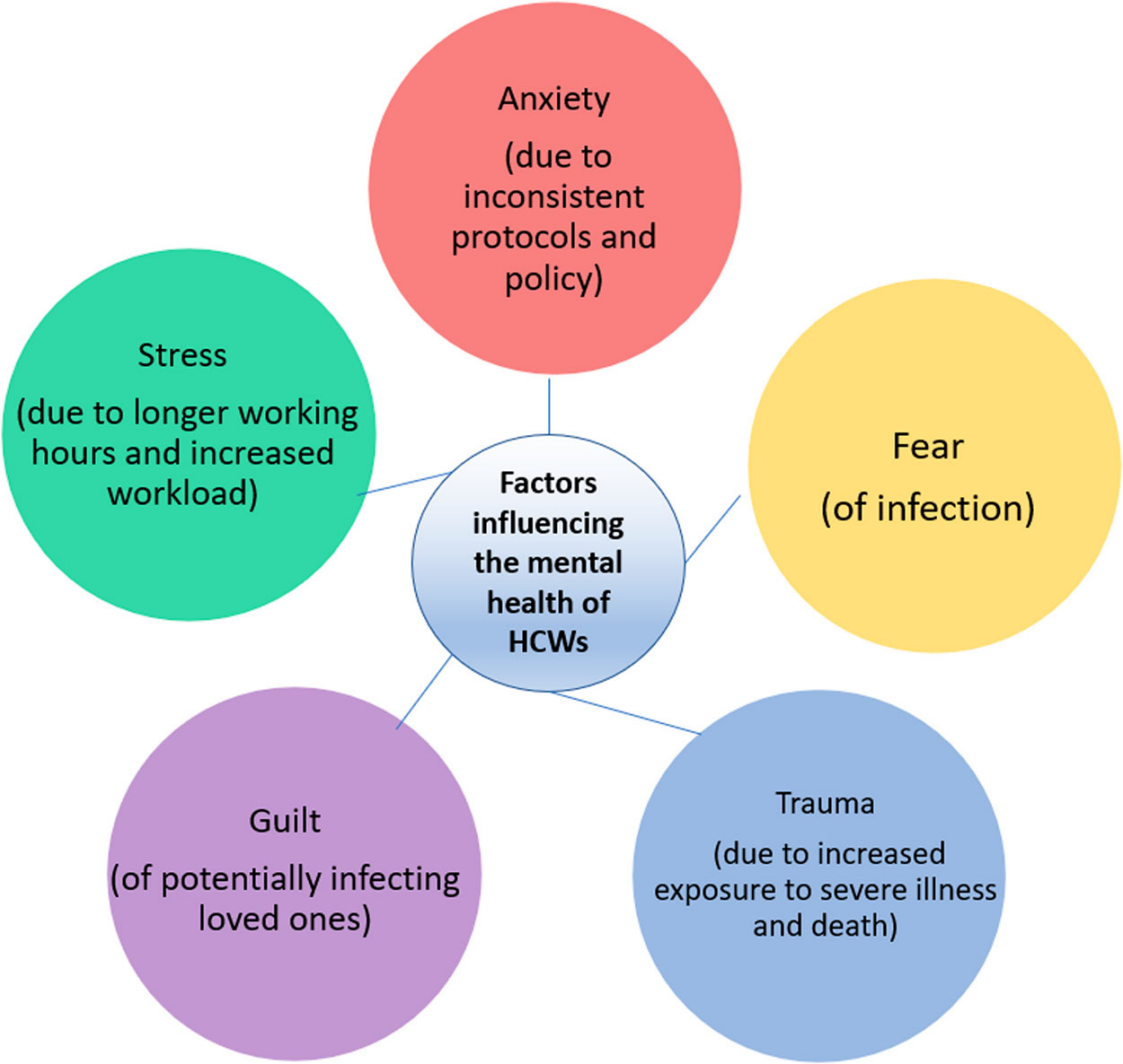
Factors influencing the mental health of all healthcare workers.

Five key themes were identified in the thematic analysis, including: anxiety (due to inconsistent protocols and policy); fear (of infection); trauma (due to increased exposure to severe illness and death); guilt (of potentially infecting loved ones); and stress (due to longer working hours and increased workload) (please see Figure 1). Each theme is presented in detail below. In addition, specific examples of recommendations for future policy and practice based on participant comments are presented in Table 2. Participant quotes are labelled by focus group number, (e.g. FG2) followed by gender and relevant HCW role.

**Table 2.** Participant based recommendations.

Participant narrative	Suggested policy recommendation
*“I think the only thing from my side would be communication … .The communication has been very confusing and, you know, lack of the right communication … . Right now the communication from the government, from the NHS, from everywhere, from your company, it’s very confusing and very little.”* (FG5, F3, Ancillary Health Worker)	Clearer, quicker and consistent communication (including policy guidance) with all staff via regular briefings utilising various media including newsletters, online/face to face, email alerts and social media
*“And for kind of future situations … .healthcare staff, especially from BAME (Black, Asian and minority ethnic) backgrounds, can be included as part of those conversations and protected because there is a risk there.”*(FG7, F, Allied Health Professional)	Inclusion of all minoritised staff groups in regular dialogue within senior management forums in order to hear their voice in relation to perceived risks and concerns
*“Yeah, so I’d like to take the flexibility forward and the inter-disciplinary working. I think it seems more levels at the moment than it has done in the past. It would be great to take that forward. More remote working and I think the advances we’ve made in technology over the past year are phenomenal when you think what the NHS hasn’t done over the past century or so”.*(FG6, F2, Allied Health Professional)	Further utilisation of new technologies to continue flexible working arrangements including remote working where possible
*“But the mental health hubs are a great idea I think. We have quite a lot of health and wellbeing at our workplace. There’s a big health and wellbeing department and they give ideas and vouchers for food and all sorts of things like that.”*(FG6, F1, Doctor)	Sustainable staff wellbeing initiatives including rewards schemes with local and national businesses, sustained access to talking therapy provision, counselling and in-house staff resources such as spaces for staff to focus on wellbeing and take self-care
*“Rather than clumping us all into one, you know, there could be some nuances between different cultures, different religions, different countries and so on.”*(FG7, M, Allied Health Professional)	More nuanced approaches to assessing staff needs, including the development and use of intersectional frameworks for risk assessments.
*“What they could do is, and I don’t know how many employees would be willing to do it, so in the pharmacy job that I’ve got in hospital, what they’ve done is with the two dispensers, they’ve split them up. It’s a financial thing for the employer. Again, it’s about capital versus, you know, livelihood versus life, where they have one dispenser coming in three days of the week one week and two days the following week, so she’s only doing five days over ten days. But she’s getting paid full time in case the other one goes down. So they never work together.” FG1, F2, Allied Health Professional*	Easing workload intensity and exposure to the virus by dividing Full Time Equivalent roles across HCWs, enabling them to work differing work patterns, potentially in different settings

### 3.3. Anxiety (due to inconsistent protocols and policy)

Participants reported feeling an increased level of general anxiety as professional practice guidance fluctuated over days and weeks, early in the pandemic. This specific confusion around ever-changing guidance for PPE and professional practice added to the general anxiety and confusion in relation to this novel virus and the global pandemic. Participants described that their anxiety levels were raised due to an implicit understanding on the part of the HCWs that employers did not actually know how to appropriately respond to the crisis. Several aspects of practice and guidance were highlighted by participants including infection control, especially during the initial phase of the pandemic:

Anxiety was the first thing. Anxiety and just not sure where we were going and just confusion. The first pandemic, it was just anxiety and confusion and didn’t really know what this meant. (FG12, F1, Doctor)

Personally I was worried, but we've always dealt with infections, but this infection was difficult, in there wasn’t clear-cut guidance around, everybody was apprehensive and felt like nobody actually really knew how to handle it at the start anyway. (FG17, M1, Doctor)

I felt like they were still navigating through the policies themselves … it’s the blind leading the blind basically at one point – that’s how it felt. (FG17, F1, Ancillary Health Worker)

Other aspects of practice and guidance where inconsistencies and resulting anxiety were described included use of Personal Protective Equipment (PPE), which was experienced across the board in healthcare settings:

We never worried about getting Covid because we were told if people were passed, they’d died, you couldn’t get it. So we didn’t, apart from wearing normal aprons and gloves and everything which you do anyway, we didn’t wear masks or anything … And then obviously new rules came in where suddenly everybody had to wear masks, even in the mortuary, and it all felt a bit too late, to be honest. (FG2, F2, Ancillary Health Worker)

The change in policies in relation to PPE seemed more prominent the first few months of 2020:

So at the beginning of the pandemic nobody – so we were office people, no PPE at all, no measures at all. After a couple of months, yeah, probably May/June or something like this then yes, they told us that we have to wear the PPE and the face masks all the time … The following week ‘you have to wear something more’, the following week ‘you have to wear something less’. So it was hard for us to reassure the staff that even with those changes. (FG5, F4, Ancillary Health Worker)

It changed all the time, like what you should be wearing and should you wear a visor, shouldn’t you? Should you wear – and people got really anxious about that. One day they were saying ‘no gloves, you have to have your sleeves rolled up’, whereas before we were wearing the gowns and people were just saying ‘I’m just not going to go in then’ … it kind of gave everyone a bit of anxiety really because you kind of thought well why are you changing the protocols all the time? (FG13, M2, Allied Health Professional)

### 3.4. Fear (of infection)

Participants were very aware of the heightened risk of infection they were facing in their roles. Ethnic minority staff were acutely aware of the disproportionate impact on them and their communities. They reported bereavements, in personal and professional capacities, and this fed into their personal fears of infection. They also questioned the rationale behind some of the decisions their employers made, which they felt put them at increased risk of exposure to the virus:

We were all kind of anxious about the virus obviously … When they called us to help out I knew OK, we were also going to be at the forefront and that meant that I would also be vulnerable to getting the virus. So the anxiety became more evident at that time, yeah. (FG12, M1, Doctor)

There was a personal fear of knowing that my colleagues within even our hub had contracted Covid, again as I said earlier, there was an unseen virus, you didn’t know where it started, you didn’t know where it was going to hit. (FG8, M3, Allied Health Professional)

The awareness of their ethnic background also impacted on how HCWs experienced fear of infection and relevant impact on their mental health during the pandemic. Some ethnic minority staff reported this explicitly:

I was actually quite worried about getting it because very early on there were signs that people from my sort of community, people of colour, were being affected quite a bit and you know, you started seeing these images coming up and they were mainly people that were from an ethnic minority. (FG1, F2, Allied Health Professional)

### 3.5. Trauma (due to increased exposure to severe illness and death)

Although many of the HCWs had experienced severe illness and death of patients as part of their work experience, the scale of illness and death and resulting psychological impact of this pandemic was clear in their responses. Again, staff from ethnic minority backgrounds reported a heightened awareness of the illness and death in their extended communities:

I felt, it was almost like being in a dreamlike state, if that makes sense, like was this happening, as such? … I sometimes think oh, I’m going to wake up and this is going to be one big horrible nightmare. (FG2, F2, Ancillary Health Worker)

Every day you leave your house and you’re going to work and it’s more or less like you are in a war zone because you’re trying to protect your life and at the same time you’ve got these very poorly patients that are gasping for relief and so you’re just drawn between two things. For me I would just describe it as if we were in a war zone and every moment is like you’re getting onto the ward and the whole atmosphere is so tense. The emotional, psychological, physical, mental exhaustion, because the time you take to see one patient, normally I would have been able to see five patients. (FG12, M2, Doctor)

The awareness of their ethnic background also impacted on how HCWs experienced trauma and relevant impact on their mental health during the pandemic. Some ethnic minority staff reported this explicitly:

Everybody who was dying seemed to be BAME [Black, Asian, and Minority Ethnic group] and it was just, I would come in and a patient that I was working on the day before and thought it was going really well and I thought my intervention, you know, I’d made some kind of difference and then I rock up the next day and they’re not there anymore and it just seemed to happen two to three times each day. (FG4, F2, Allied Health Professional)

One participant reported being redeployed into a role where they faced exposure to death on a daily basis, having never worked in a clinical capacity before:

So I came from a position of never being clinical, never seeing somebody that had passed away, because I literally, I actually had a fear of dead bodies before I went and worked in the mortuary … Seeing all that death and it was literally I woke up one day and I was like ‘all I see is death. All I see is death. There’s no life.’ And I think that was what got me after a while. (FG2, F2, Ancillary Health Worker)

Some participants reported the cumulative psychological impact of the illness and related bereavement in both professional and personal capacities. The scale as well as personal connection with loss and death seemed to be a clear factor impacting on their mental state. Professional examples included:

I remember the reaction of the second Covid positive when I was about to approach him and he stopped me and he said, ‘Don’t come near me because I have the virus,’ and he was in tears, he was crying. (FG3, F, Nurse)

I had a [teenager] die in front of me because, again, you know, keeping it anonymous but [they] had [a comorbidity] and when you have [it] your impact of the Covid infection is literally a different level … And [they] were well until they weren’t and [they] died very quickly, as in [they] deteriorated very fast. (FG8, M1, Doctor)

Personal loss was also discussed by the participants:

I’ve lost 16 friends already with Covid and I don’t know if I’m still counting and I don’t want to count any more because I think it’s just relentless. (FG3, F3, Nurse)

### 3.6. Guilt (of potentially infecting loved ones)

Many participants spoke of the guilt they felt regarding potentially infecting their family and loved ones. They were very aware of the heighted risk of infection they faced due to their professional role, but felt they were then passing on that risk to those that they lived with. This guilt had a clear impact on their mental wellbeing:

For me it was that guilty feeling, and still is, that I am the one who’s going to kill somebody because I’m going into an area where I know there’s Covid patients and then coming back to people who could actually stay pretty safe, if it wasn’t for me … I felt like I was some kind of lethal weapon walking around! (FG15, F2, Nurse)

If I have to die, let me die with a heart attack rather than Covid positive and I bring that back and infect my children … Not only scared for myself, again, but probably as we all felt, we are more scared for our family than ourselves. I was extremely scared for my husband … now if I have to go somewhere within the hospital, I avoid it. If I don’t have to, I don’t. I really avoid going out. I’m scared. I’m scared. (FG5, F3, Ancillary Health Worker)

I’m then putting my family and friends at risk. I feel like I’m the one that is the danger to everybody now. (FG15, F2, Nurse)

### 3.7. Stress (due to longer working hours and increased workload)

A number of HCWs reported having to work longer hours during this period, describing the increased workload and expectation that they worked beyond their contracted hours:

So my work pattern now should be 9 to 5. Obviously most of us aren’t working those at the moment. So at the moment it’s about 7 to 8. (FG2, F1, Nurse)

You’re supposed to be just working Monday to Friday 8 to 5 or 7 till 4, but sometimes we’ll go in at 7 and at 10 o’clock I’m still there. (FG3, F1, Nurse)

We had two teams working there and I was there from 7 till 11 at night, basically looking after one team and we did everything and I worked 14 days on, 2 days off. It was just – and like weekends, there was no break, there was no break from it. (FG2, F2, Ancillary Health Worker)

The awareness of their ethnic background also impacted on how HCWs experienced stress during the pandemic. Some ethnic minority staff reported this explicitly:

For me, the biggest problem was that the department I work in, the majority of us are from ethnic minority groups. (FG5, M3, Ancillary Health Worker)

I was one who was kind of watching the news and just seeing how things were developing and every day watching the news and seeing this sort of role-call of clinicians who were dying and, unfortunately, they were all looking like me. And I became very concerned about what I was seeing and, as now has been acknowledged, a disproportionate effect that it was having on the ethnic community. (FG8, M3, Allied Health Professional)

Participants described how they felt that they had an individual responsibility to help cover the increase in demand in patient care, however this came at a cost for them in terms of increased stress, pressure, and fatigue:

I would literally be in tears because I cannot cope with the stress anymore. I cannot cope with they want this, this, this and they want it yesterday. I can’t do it. (FG5, F3, Ancillary Health Worker)

You know, each day of the week we go to work we’re carrying baggage on our backs, a baggage of stress, of anxiety, of saying ‘make sure I do it right.’ (FG12, F, Doctor)

Healthcare organisations lacked the capacity to deliver usual levels of care. The increase in work included the diversity of responsibilities HCWs took on, as well as the increase in patients that each of them was expected to care for:

I was doing everything. I was doing management, I was running the ward, I was teaching, because so many people in our ward, they were actually ill or shielding, which is understandable, so they were not around. (FG3, F2, Nurse)

I came back to work after being unwell and then a few days later it was just me with (nearly 30 patients) - 4 of them were (intubated) – because everyone else was unwell. (FG3, F2, Nurse)

The reporting of chronic understaffing was coupled with the direct impact on HCWs and how many patients they had to care for. The mental strain of this was apparent in their testimonies:

It’s normally one to one nursing but there were some days when it was one to six, one nurse to six patients, with an average of say four or five. So the nurse was just running around. So we were just keeping an eye on the patients, washing them, doing the ventilators, suctioning, that kind of thing, eye care – just all those kind of little bits that I actually found out later are actually a really important part of intensive care. (FG13, M2, Allied Health Professional)

Three or four staff who had to shield because of … different conditions, so it was just very very difficult … Normally we’d have about 280 patients on the caseload and there were 380. (FG6, F1, Doctor)

## 4. Discussion

In a nationwide study of HCWs from a range of occupations (clinical and non-clinical) and from diverse ethnic backgrounds, we undertook an in-depth exploration of their experiences while working during the pandemic, and the impact of this on their mental health. Five key themes were identified which impacted on the mental health of healthcare workers from across diverse ethnic backgrounds during this period including anxiety (due to inconsistent protocols and policy); fear (of infection); trauma (due to increased exposure to severe illness and death); guilt (of potentially infecting loved ones); and stress (due to longer working hours and increased workload). The findings from this research generally align with the emerging evidence base that there is an increased psychological burden on HCWs during the COVID-19 pandemic and this is likely due to the staff reorganisation, the working intensity, and the anxiety of being exposed to the virus in the healthcare setting and, in turn, of bringing the infection home (Di Tella, Romeo, Benfante, & Castelli 2020). Other research also points to increased hours and exposure to COVID-19 patients as factors contributing to poorer mental health among HCWs (Woolf et al., 2021), in line with our findings.

However, the findings from this research also suggest that organisational responses to the pandemic were inconsistent. There was a distinct absence of well-defined and consistent policy in regard to pandemic planning and preparedness. The lack of clear and consistent guidance left HCWs at risk of physical as well as mental harm. Our research shows that HCWs were acutely aware of this increased risk. People with different roles within the healthcare workforce were provided with different guidance and different levels of protection. This included (lack of) access to PPE. Some research suggests that NHS staff were put at risk due to insufficient delivery and provision of appropriate PPE, reporting that by April 2020, only 12% of hospital doctors felt fully protected from the virus at work (Scally, Jacobson, & Abbasi, 2020).

In addition to the shared experiences described across our diverse sample, the research also sheds light on some specific circumstances which relate to the experience of ethnic minority HCWs in particular. These findings align with other research (Martin et al., 2022) which identified less access to adequate PPE for ethnic minority HCWs. This situation is associated with decreased trust in employers (as evidenced in this research), which in turn may be associated with a more significant impact on the mental wellbeing of ethnic minority HCWs compared to those from white ethnic backgrounds (Melbourne et al., 2022). Inconsistencies in policy guidance may leave HCWs at increased risk of infection from COVID-19, and combined with a lack of trust in employer, may lead to a fundamental sense of betrayal. This loss of trust and sense of betrayal has been measured in previous research (e.g. using the moral injury event scale Lamb et al., 2021), and provides further evidence of the detrimental psychological impact on the mental health of HCWs during COVID-19. Whilst this was experienced across a diversity of ethnic groups, the findings suggest this can exacerbate existing marginalisation and inequities among staff from ethnic minority backgrounds in particular (Lamb et al., 2021). These findings need to be considered in the specific circumstance of some ethnic minority staff facing racial discrimination (Mistry & Latoo, 2009; Likupe & Archibong, 2013; Thorne, 2017; Qureshi, Garcia, & Ali, 2021) and the cumulative effect that experience can have on the mental health of ethnic minority people (Schmitt, Postmes, Branscombe, & Garcia, 2014; Pascoe & Richman, 2009).

Furthermore, the findings in this research highlight a wider potential gap in national policy responses to COVID-19. Historically, a number of government commissioned reports have placed patient safety at the heart of quality of healthcare service provision (Darzi, 2008; Colin Thomé, 2009). However, it seems that when responding to COVID-19, the government’s primary strategy was to retain the required critical care capacity during surges in demand (McCabe et al., 2020). This meant that existing problems like the longstanding shortage of nurses and other HCWs in the NHS (Buchan & Seccombe, 2013) may have thus negatively impacted on patient safety and staff to patient ratios during COVID-19, as evidenced in our findings. Although the NHS has consistently resisted the imposition of minimum staffing ratios (Lawless, Couch, Griffiths, Burton, & Ball, 2019), in 2019 guidelines from the Faculty of Intensive Care Medicine (FICM) and Intensive Care Society (ICS) described the levels of care required by critically ill patients in hospital according to their clinical needs. The guidance was clear that ventilated patients must have a registered nurse/patient ratio of a minimum 1:1 to deliver direct care (Hill, 2020). As the second wave of COVID-19 hit the UK in Winter of 2020, National Health Service (NHS) England advised hospitals to temporarily suspend the 1:1 ratio for level 3 critically unwell patients (Hill, 2020). The findings in this paper show the impact of that decision on HCWs and their mental well-being Participants in our research across ethnic groups reported how these national decisions resulted in extremely challenging increases in workload and working hours for these HCWs.

**Figure 2.**
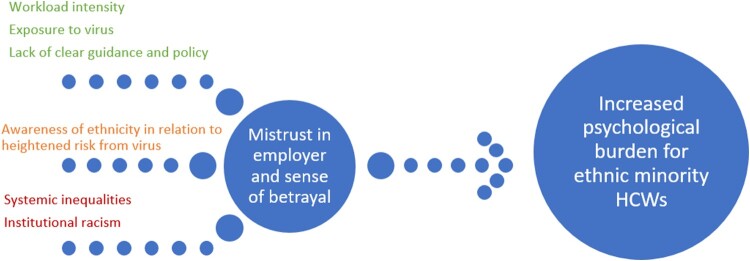
Cumulative effect on ethnic minority healthcare workers. Contributary factors highlighted in green may be applicable to most HCWs; factors in amber apply to ethnic minority staff; those in red are long standing factors impacting on ethnic minority HCWs. The cumulative impact of all factors can lead to an increased mistrust in employer and sense of betrayal resulting in increased psychological burden for ethnic minority HCWs.

The emerging evidence base suggests that ethnic minority communities are more likely to experience poor health outcomes and higher mortality rates due to COVID-19 (Nafilyan et al., 2021). Our findings demonstrate that across diverse ethnic groups, HCWs are experiencing mental distress and trauma due to experiencing significant amount of loss and increased exposure to death at work and bereavement of friends and family. This is likely to exacerbate existing structural and systemic inequities, and disparities in the impact of COVID-19 within the health system. Furthermore, for HCWs from ethnic minority backgrounds, these occupational stressors are likely to be compounded with the wider loss and bereavement they are experiencing at the intersections of their professional and personal communities due to COVID-19. Despite consistent evidence that people of minority ethnicity backgrounds are at higher risk of catching COVID-19 and dying from it (Razaq et al., 2020; PHE, 2020b), and the increased risk of exposure HCWs from these communities face due to their frontline roles, few solutions have been communicated or actioned (Mutambudzi et al., 2021; Zhao, He, Xie, & Liu, 2020). This paper suggests some recommendations based on participant quotes (Please see Table 2) which include: Clearer, quicker and consistent messaging utilising various media; Inclusion of all minoritised staff groups in regular dialogue within senior management forums; Further utilisation of new technologies to continue flexible working arrangements; Sustainable staff wellbeing initiatives; and more nuanced approaches to assessing staff needs and easing workload intensity and exposure to the virus by dividing Full-Time Equivalent roles across HCWs. The findings in this paper also evidence the cumulative effect of these relative risks on the mental health of HCWs (please see Figure 2).

## 5. Limitations

Due to social distancing measures in place at the time, recruitment strategies and data collection had to be conducted remotely and using online technology. This may have affected participation from certain groups who may be less proficient in use of or have less access to digital technology. The study data was collected in real-time as the situation around the COVID-19 pandemic was unfolding may be seen as a strength in terms of relevance but may also mean that some of the participants’ views may have changed from the time of data collection, as relevant policies and hospitalisation and infection rates varied considerably over that time period. Participants may have been at risk of recall bias during interviews. Views may have also been influenced by outside data sources and policies introduced. In addition, the broad age range of the sample may have impacted results, and this too could be considered a limitation, though it also provides important nuance and insight into the experiences of the diverse healthcare workforce given the large age ranges present in the NHS as well.

## 6. Conclusion and recommendations

This paper highlights some of the prominent factors influencing the mental health and emotional wellbeing of HCWs from diverse ethnic backgrounds during the pandemic (see Figure 1). Our findings align with the emerging evidence base, which has highlighted staff reorganisation; working intensity; being exposed to the virus; bringing the infection home; and increased hours and exposure to COVID-19 patients as factors impacting the mental health for HCWs. However, our study also highlights the lack of clear local and national policy and preparedness, and lack of access to PPE and other factors resulting in higher psychological burden for HCWs. Furthermore, the intersectional impact (Crenshaw, 1991) of belonging to an ethnic minority leading to a heightened sense of awareness of the link between ethnicity and higher exposure to death and severe illness, personally and professionally in addition to experiencing systemic inequality and potential decrease in trust and sense of betrayal may also contribute to a higher psychological burden for ethnic minority staff (please see Figure 2). The findings in this paper add to the existing evidence base by providing perspectives from an ethnically diverse workforce, which can be interpreted and used by individual healthcare organisations in their individual contexts. However, Table 2 below provides some potential examples of future policy and practice based on participant recommendations. Further to these, we recommend that healthcare organisations should consider the specific circumstances of HCWs, and inequities among staff, to develop strategies to counter the negative impact of these factors on mental health.

## Declaration of interests

MP reports grants from Sanofi, grants and personal fees from Gilead Sciences and personal fees from QIAGEN, outside the submitted work. IQ, MG, FW, IQ, AAO, JC OH and LBN have no competing interests to declare.

## Data Availability

To access data or samples produced by the UK-REACH study, the working group representative must first submit a request to the Core Management Group by contacting the UK-REACH Project Manager in the first instance. For ancillary studies outside of the core deliverables, the Steering Committee will make final decisions once they have been approved by the Core Management Group. Decisions on granting the access to data/materials will be made within eight weeks. Third party requests from outside the Project will require explicit approval of the Steering Committee once approved by the Core Management Group. Note that should there be significant numbers of requests to access data and/or samples then a separate Data Access Committee will be convened to appraise requests in the first instance.

## Appendix TOPIC GUIDE AREAS

### Topic Guide Areas

**Table:**

**OBJECTIVES** • To gather reflections on participants’ views and experiences as clinical and non-clinical healthcare workers during the COVID-19 pandemic; • To explore what factors healthcare staff feel have put them or other healthcare staff at risk; • To examine fears or concerns healthcare staff have experienced both in and outside of work; • To discuss challenges healthcare staff have experienced in accessing the information they need for protecting themselves; • To identify things that have been helpful in their workplace or community in supporting healthcare staff or protecting their health.

**Table:**

Heading	Examples of subject matter
Introduction	Project overview, consent information etc
Participant background information	Biographical data
Fears, concerns and perceived risk factors	Individual level factors
Challenges relating to accessing information or ability to protect yourself	Experiences in accessing information
Support and facilitators	Coping mechanisms
Cultural, ethnic and migration related factors	Influence of culture
Stigma and discrimination	Impact of stigma
Protection and promotion	Recommendations for the future
Closing	Thanks and final opportunity to contribute any more thoughts/comments/suggestions
	
